# Complete Genome Sequence of Enterococcus faecium QU50, a Thermophilic Lactic Acid Bacterium Capable of Metabolizing Xylose

**DOI:** 10.1128/MRA.00413-19

**Published:** 2019-05-23

**Authors:** Kiyotaka Abe, Yu Kanesaki, Mohamed Ali Abdel-Rahman, Satoru Watanabe, Takeshi Zendo, Taku Chibazakura, Mariko Shimizu-Kadota, Kenji Sonomoto, Hirofumi Yoshikawa

**Affiliations:** a Department of Bioscience, Tokyo University of Agriculture, Tokyo, Japan; b Research Institute of Green Science and Technology, Shizuoka University, Shizuoka, Japan; c NODAI Genome Research Center, Tokyo University of Agriculture, Tokyo, Japan; d Botany and Microbiology Department, Faculty of Science (Boys), Al-Azhar University, Cairo, Egypt; e Laboratory of Microbial Technology, Division of Systems Bioengineering, Department of Bioscience and Biotechnology, Faculty of Agriculture, Graduate School, Kyushu University, Fukuoka, Japan; f Department of Environmental Systems Sciences, Musashino University, Tokyo, Japan; Portland State University

## Abstract

Herein, we report the complete genome sequence of Enterococcus faecium QU50, isolated from Egyptian soil and exhibiting intermediate susceptibility to vancomycin. The genome contains a 2,535,796-bp circular chromosome and two plasmids of 196,595 bp and 17,267 bp. IS*1062*-like sequences were not found.

## Abstract (precis)

Herein, we report the complete genome sequence of Enterococcus faecium QU50, isolated from Egyptian soil and exhibiting intermediate susceptibility to vancomycin. The genome contains a 2,535,796-bp circular chromosome and two plasmids of 196,595 bp and 17,267 bp.

## ANNOUNCEMENT

Enterococcus faecium QU50, isolated from Egyptian soil, has potential use in the industrial production of poly-l-lactic acid from biomasses because of its superior abilities to metabolize xylose in a homolactic fermentation manner and to grow at 50°C (1, 2). In addition, QU50 has an intermediate susceptibility to vancomycin (MIC, 4 μg/ml) and resistance to erythromycin (MIC, >100 μg/ml). For a better understanding of QU50 and its evolutionary position within the species, known as vancomycin-resistant enterococci, we have deciphered its complete genome sequence.

QU50 was isolated as described in detail previously (1), and its genomic DNA was prepared according to the protocol followed for Enterococcus mundtii QU25 (3). Sequencing was conducted on a PacBio RS II sequencer at TaKaRa Bio, Inc. (Kyoto, Japan), using two single-molecule real-time cells with a 10-kb insert library with P5-C3 chemistry. The 254,043 reads obtained with a total of 491,869,404 nucleotides (192× coverage) were assembled *de novo* using RS_HGAP Assembly.2 with default settings and a seed read cutoff length of 10,000 bp to form three contigs. The number of postfiltered reads was 76,548 with a read quality of 0.84. By a similarity search using the ATSQ program in GENETYX-MAC v.18.0.0 (Tokyo, Japan) and *in silico* Molecular Cloning Genomics edition v.6.0.0F (In Silico Biology, Inc., Yokohama, Japan), we found both terminal regions of each contig overlapping. PCR amplifications were performed using the following primer pairs, whose directions are outward to both ends: 5′-CAAACAATCAACACGGAACCAACC-3′ and 5′-CATGGGCATCCAGGAGATTATCAATTTC-3′ for the largest contig, 5′-CGGAATTAGTCCCAGAAGACCAATG-3′ and 5′-CGTTCACTGGATTGACCAGTCAG-3′ for the middle-sized contig, and 5′-CTTTTCACTCTAGCGGTTGGAGG-3′ and 5′-GCAGCCCATTCAGCGGG-3′ for the smallest contig; then, we obtained 15 kbp, 10 kbp, and 1.6 kbp fragments, indicating that the sequences of these contigs form in three circular patterns. Therefore, we removed the overlapping regions from each of the contigs by manual curation and finally obtained three circularized sequences, which corresponded to a chromosome and two plasmids. Their sequences were corrected by comparison with those obtained using Illumina MiSeq (362× coverage) and those from manual capillary sequencing. MiSeq sequencing was performed using the MiSeq reagent kit v.3 (600 cycles). A sequencing library was generated using the NEBNext DNA library prep kit (NEB). Read mapping to the circular genome was performed using the CLC Genomics Workbench v.7.5 (Qiagen) with the following parameters: read quality, phred score >30; trimming, 5′ cutoff of 15 bp and 3′ cutoff of 2 bp; mismatch cost, 2; indel cost, 3; length fraction, 0.8; and similarity fraction, 0.9. From the data obtained, we found only one error in the original RS_HGAP Assembly.2.

The QU50 genome comprises a chromosome of 2,535,796 bp (GC content, 38.42%) and two plasmids, pQL50 (196,595 bp; GC content, 35.66%) and pQS50 (17,267 bp; GC content, 39.11%). Annotations and other analyses were performed using DFAST v.1.1.4 (4) with default settings. On the chromosome, two clear GC skew shift points were found around the start codon of *dnaA* and the 28-bp *dif* sequence at the positions of 0% and 53.44%, respectively. In total, 2,405 protein-coding DNA sequences (CDSs), 68 tRNA genes, and 6 rRNA operons were predicted. pQL50 contains 192 CDSs and 1 tRNA gene. pQS50 has no RNA genes and 25 CDSs, including an erythromycin resistance gene, since pQS50-cured derivatives exhibited higher sensitivity for erythromycin (MIC, 2 μg/ml). These were able to be isolated after the introduction of a plasmid vector, pGKV11 (5), to QU50, possibly because these plasmids are incompatible with each other.

Strain-specific genes for producing putative xylulose 5-phosphate from xylo-oligosaccharide (e.g., xylose isomerase, xylulokinase, xylose operon repressor, and xylan 1,4-beta-xylosidase) were localized at 910,584 to 921,538 bp, with a higher GC content of 41.01% resulting possibly from horizontal gene transfers. Genes responsible for homolactic fermentation of pentoses (i.e., transaldolase and transketolase) and those for heterolactic fermentation (i.e., phosphoketolase, acetate kinase, phosphotransacetylase, and alcohol dehydrogenase) were also annotated.

PHAST (http://phast.wishartlab.com/; latest version, 15 March 2016) identified three independent prophage-like sequences on the QU50 chromosome and on pQL50, showing that large-scale inversions are unlikely to be caused by these sequences (6, 7). DFAST identified 186 insertion-like sequences on the QU50 chromosome and on pQL50. However, the QU50 genome does not have the IS*1062*-type copies, especially those downstream of the genes for vitamin B_12_-independent methionine synthase and for GTP-binding protein HflX, in which large-scale genome inversions have sometimes occurred in E. faecium strains (6, 7).

This complete genome sequence of QU50 will contribute to elucidation of the epidemic derivation of E. faecium strains within the species through comparison of their genome structures (8). This information is also useful for establishing the optimal traits of QU50 for efficient l-(+)-lactic acid production from lignocellulose biomasses containing xylose through homolactic fermentation.

### Data availability.

The complete genome sequences for E. faecium QU50 were deposited in GenBank/ENL/DDBJ under the accession numbers AP019394, AP019395, and AP019396. The versions are all the first versions. The accession numbers of the original read data set in the SRA are DRR140425, DRR140426, and DRR140427.
